# Barriers and challenges of infant feeding in disasters in middle- and high-income countries

**DOI:** 10.1186/s13006-021-00398-w

**Published:** 2021-08-23

**Authors:** Cindy H. Hwang, Alessandro Iellamo, Mija Ververs

**Affiliations:** 1 Johns Hopkins Bloomberg School of Public Health, Baltimore, MD USA; 2 Save the Children, London, England; 3 Johns Hopkins Bloomberg School of Public Health, Center for Humanitarian Health, Baltimore, MD USA

**Keywords:** Nutrition in emergencies, Infant feeding, Breastfeeding, Disaster planning, Emergency preparedness

## Abstract

**Background:**

Global evidence from the past 35 years confirmed the protective effect of breastfeeding and supported the guidance to protect, promote, and support breastfeeding practices, particularly in natural disaster and conflict settings. This study aimed to summarize the difficulties faced by disaster responders and mothers for optimal infant feeding during disasters in middle and high-income countries.

**Methods:**

A scoping literature review was conducted by searching databases for peer-reviewed literature and grey literature published between January 2010 and December 2018 that focused on infant feeding in the aftermath of disasters. Only disasters that occurred in middle or high-income countries as defined by the World Bank for the 2018 fiscal year were included.

**Results:**

The study found that a major challenge faced by organizations establishing infant feeding in emergencies (IFE) programs is the violation of The International Code of Marketing of Breastmilk Substitutes by other aid organizations and governments, such as acceptance of donated infant formula and untargeted distribution of formula. Additionally, many disaster responders were unfamiliar with IFE protocols. Mothers faced other barriers to breastfeed their infants during disasters. They often lacked privacy or spaces conducive to breastfeeding. Limited fluid and energy intake, stress, and exhaustion also deterred mothers from breastfeeding. Many challenges for responders and barriers mothers face for optimal infant feeding practices persist despite existing guidelines.

**Conclusions:**

The findings of this study reveal the lack of IFE preparedness and response capacity in middle and high-income countries, and the need for governments and aid organizations to adapt guidelines and establish policies and programs to support infant feeding in emergencies.

## Background

Breastfeeding is the most cost-effective intervention to improve the health and development of both children and women. A recent meta-analysis confirms previous findings that breastfeeding protects children from infection mortality and morbidity, increases intelligence, and reduces risk for diabetes [1, 2]. For mothers, breastfeeding can prevent breast cancer, improve birth spacing, and may reduce risk for diabetes and ovarian cancer [1].

Global evidence confirmed that the protective effect of breastfeeding for infants is particularly important during natural disasters and conflict settings. After the Bosnian conflict, a study using cross-sectional household surveys from 1994 to 1997 revealed that non-breastfed children were more likely to be malnourished [3]. During the 2006 floods in Botswana, infants hospitalized with diarrhea were 30 times more likely not to have been breastfed compared with infants without diarrhea [4]. After the 2006 earthquake in Indonesia, the use of donated infant formula doubled the rates of diarrhea in young children compared with those who had not received donated infant formula [5]. In the aftermath of the 2005 Hurricane Katrina that flooded the city of New Orleans in the United States, several infants died of dehydration when food and water supplies ran out due to a fractured coordination of disaster response [6].

For more than 35 years, growing global evidence has supported the dissemination of guidance and recommendations to help governments set up conditions and systems to protect, promote, and support breastfeeding practices. Table 1 shows key documents published or supported by United Nations (UN) agencies and international organizations [7–13]. In 1981, The International Code of Marketing of Breastmilk Substitutes was endorsed and set standards on the marketing and promotion of breastmilk substitutes (BMS), including infant formula [7]. Nineteen subsequent World Health Assembly (WHA) Resolutions updated and added recommendations and standards as scientific evidence mounted supporting breastfeeding practices and concerns surrounding infant formula feeding [8]. The 1981 resolution and subsequent relevant WHA resolutions will be referred to as ‘The Code’ in this study. The Operational Guidance for Infant Feeding in Emergencies, developed by the Infant Feeding in Emergencies (IFE) Core Group, outlines key provisions that should be included in government and agency policies for emergency responses. The guidance emphasizes the importance of training personnel across sectors for IFE response, calls for governments to ensure capacity to coordinate IFE efforts and provides recommendations that meet international standards for infant feeding to protect, promote and support breastfeeding and to minimize the risk of artificial feeding [11].

**Table 1:**
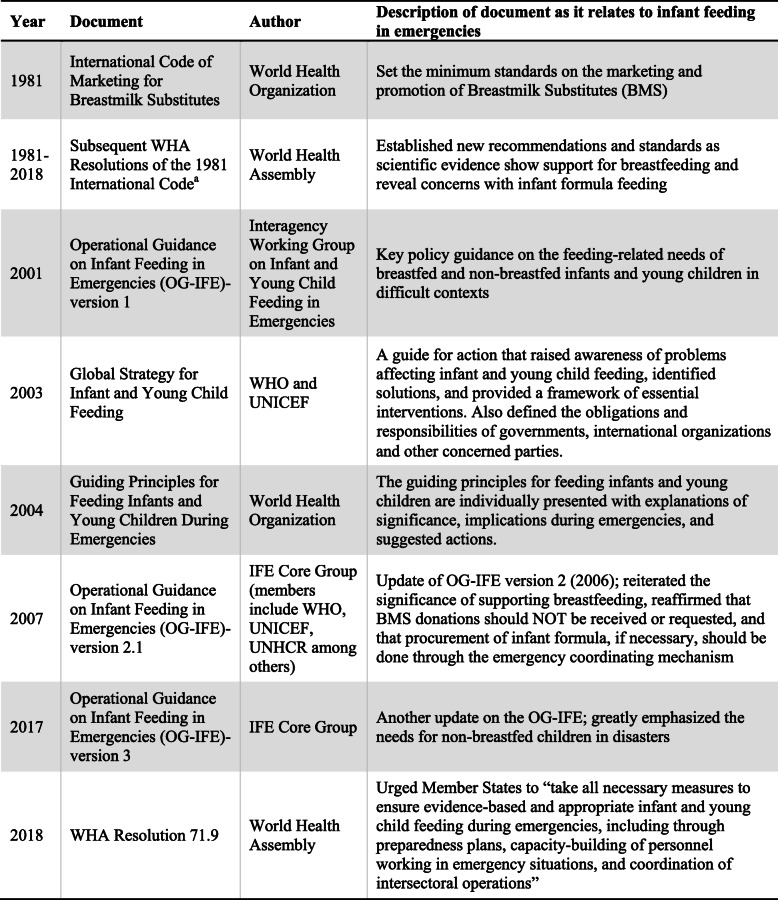
Key documents providing guidelines and recommendations for infant feeding in emergencies

^a^WHA Resolutions 35.26, 37.30, 39.28, 41.11, 43.3, 45.34, 46.7, 47.5, 49.15, 54.2, 55.25, 58.32, 59.11, 59.21, 61.20, 63.23, 65.6, 69.9, 71.9

In 2018, the WHA endorsed Resolution 71.9, which urged Member States to “*take all necessary measures to ensure evidence-based and appropriate infant and young child feeding during emergencies, including through preparedness plans, capacity-building of personnel working in emergency situations, and coordination of intersectoral operations*” [12]. Despite existing global guidance and evidence-based recommendations, only 25% of Member States have IFE protocols within their national nutrition policies [14].

Even in more developed middle-income (MICs) and high-income countries (HICs), inadequate disaster response negatively impacts upon appropriate infant feeding practices, leaving infants at risk of serious health and developmental consequences. This review aimed to present key issues and concerns related to IFE in MICs and HICs during disaster response focusing on 1) the challenges of IFE program implementation for responders, and 2) the barriers to breastfeeding that mothers face during disasters.

## Methods

A literature review was conducted from 3 January to 1 February 2019 using PubMed and SCOPUS, with citation searching of relevant articles. Key phrases and Medical Subject Heading terms used in searches included “infant feeding in emergencies”, “infant nutrition in emergencies”, “breastfeeding during emergencies” and “refugee infant nutrition”. Searches using “disasters” in place of “emergencies” also yielded similar results. Additional searches of the grey literature and news reports were conducted using Google and reviewing Field Exchange, the technical publication of Emergency Nutrition Network. The same key phrases used for the grey literature search were applied to the search of the databases. Both searches were conducted by one author with guidance from the co-authors.

**Fig. 1:**
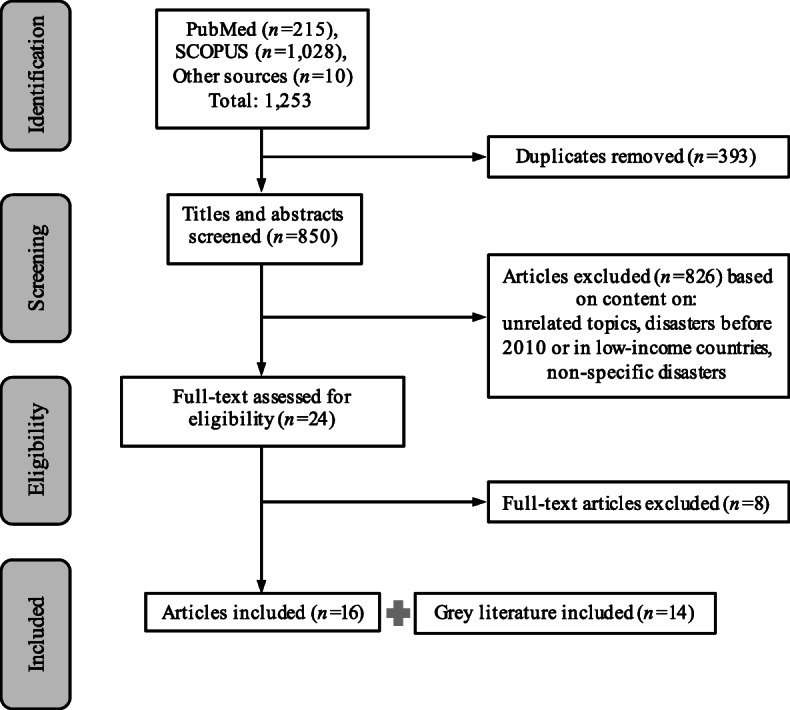
Flow diagram of literature search and selection process

Articles were selected for inclusion if they: 1) were published between January 2010 and December 2018 and focused on disasters, 2) occurred in middle or high-income countries as defined by the World Bank for the 2018 fiscal year [15] and 3) described IFE programs in specific disasters. Only articles in English were included. Articles were excluded if they focused on unrelated topics, disasters occurring before 2010 or in low-income countries, or did not focus on a specific disaster (Fig. 1).

The data was extracted by one author using a prepared electronic form. The information collected included the setting (location, year and type of disaster), type of article, and a summary of infant feeding interventions and/or programs provided during a disaster. Following the data extraction, a narrative synthesis was undertaken by two authors. A preliminary synthesis tabulated the findings which were then discussed and structured into the two major themes presented in this review. Within the two main themes, common findings in both conflict and natural disaster settings are presented, followed by findings unique to each context to highlight the differences between the two. The study focuses on MICs and HICs because research on infant feeding tends to focus on low- and middle-income countries; the findings from MICs and HICs were analyzed as a unit and were not compared.

For the purposes of this study, a disaster or emergency is defined as an event or series of events where human, material, economic or environmental damage critically threatened the health, safety, security or wellbeing of a community or a region, and exceeded the community’s capacity to cope [16, 17]. For the purpose of this study, emergencies are classified into two groups: 1) natural disasters, which include geophysical, hydrological, climatological, meteorological and biological disasters, or 2) conflicts such as situations impacted by political unrest and armed conflict.

## Results

A total of 30 articles representing 17 countries were included in the review. The characteristics of these articles are presented in Table 2. The articles reviewed included: research studies [18–26], field reports [27–36], perspective papers [37–45] and news releases [46, 47]. Almost half of the reviewed articles focused on emergencies in Europe and the Middle East, most of which were related to the 2015 European migrant crisis. Eight articles focused on natural disaster-related emergencies in other parts of the world, with four occurring in Asia, two in North America and one in Africa and Oceania, respectively. Natural disasters included earthquakes, hurricanes, floods, droughts and wildfires. A total of 15 articles were natural disaster-related and 15 articles were conflict-related.

**Table 2:** Characteristics of reviewed articles (*n* = 30)

Characteristics of articles				No. of articles
Country/Territory*		Canada		2
(*n* = 17)		Croatia		2
		France		1
		Greece		4
		Indonesia		1
		Iraq		2
		Japan		3
		Jordan		2
		Kenya		1
		Lebanon		2
		Macedonia		1
		Malaysia		1
		New Zealand		1
		Pakistan		5
		Puerto Rico		2
		Serbia		2
		Ukraine		3
Country Income Status*		High		15
		Upper middle		10
		Low middle		10
Setting		Conflict		15
		Natural Disaster		15
Article Type		Research		9
		Field report		10
		Perspective		9
		News Release		2

*Some articles discussed more than one country

The findings of the literature review are presented under two main themes: 1) challenges faced by disaster responders that hindered appropriate IFE program implementation, and 2) barriers faced by mothers (reported and observed) related to breastfeeding and/or provision of breastmilk for infants in these settings. The challenges associated with IFE program implementation impacted the feeding practices mostly for infants, as children < 1 year old as defined by the World Health Organization (WHO) [48], however, the breastfeeding barriers mothers faced during disasters impacted both infants and children > 1 years old.

### Challenges of appropriate IFE program implementation for responders

#### Context of both conflicts and natural disasters

The literature review revealed the many challenges of IFE program implementation during conflict and natural disasters (Table 3). In every situation, The Code was violated. Most common violations involved the acceptance of infant formula donations and untargeted distributions of infant formula [18, 20, 24–30, 32–40, 43, 44, 46, 47]. In many instances, health and nutrition responders lacked the knowledge, or were not willing to follow established IFE protocols, all issues which undermined any support for appropriate infant feeding practices [18, 24, 26, 27, 30, 32, 33, 36, 42–44]. The high pre-crisis mixed-feeding (feeding of breastmilk and any other foods or liquids) rate of many affected populations and their preference for infant formula contributed to the difficulty of protecting, promoting and supporting breastfeeding as the safest and healthiest method of feeding infants [23, 25, 26, 30, 32, 35, 36, 44, 46, 47]. Another major challenge involved breastfeeding misconceptions among mothers and families which often led to their decision not to pursue breastfeeding [25, 26, 32, 35, 36, 44, 47]. Other frequently cited common challenges included a lack of coordination, support, expertise, and physical spaces to provide IFE services [24, 26, 27, 30, 33, 34].

**Table 3:**
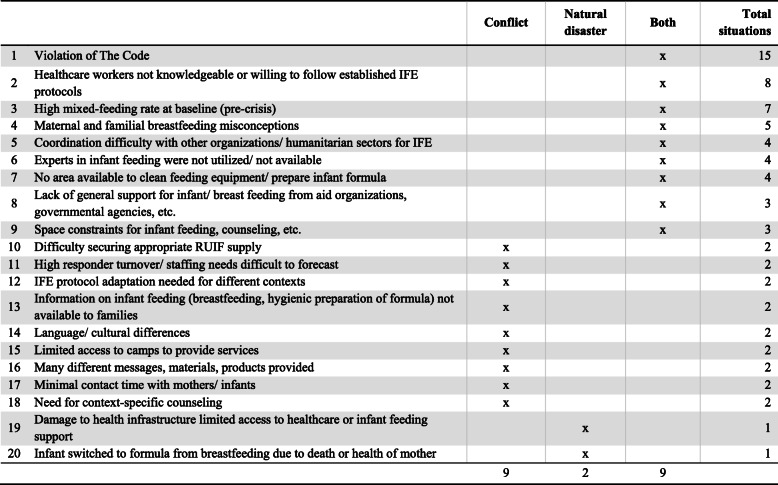
Summary: Challenges of IFE program implementation for responders, by type of emergency (*n* = 28)

The Code, The International Code of Marketing of Breastmilk Substitutes and subsequent WHA resolutions

IFE, infant feeding in emergencies

RUIF, ready-to-use infant formula

#### Context of conflicts

Table 4 shows all 18 challenges that hindered implementation of appropriate IFE services by responders in conflict-related settings. Nine of these challenges were unique to the conflict setting (Table 3). While some challenges were encountered in most situations, different conflicts also presented with their own challenges.

**Table 4:**
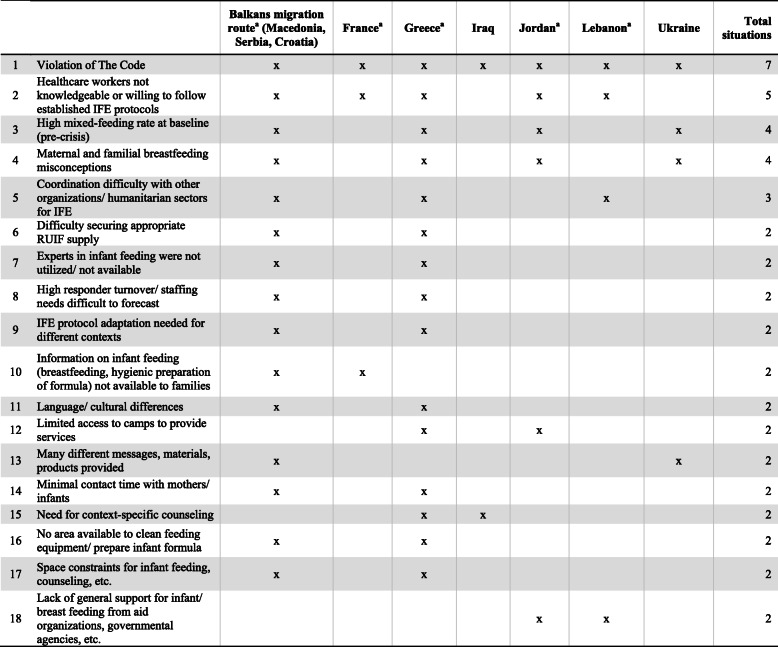
Challenges of IFE program implementation for responders due to conflicts, by geographical area (*n* = 15)

The Code, The International Code of Marketing of Breastmilk Substitutes and subsequent WHA resolutions

IFE, infant feeding in emergencies

RUIF, ready-to-use infant formula

^a^These countries were affected by the European migrant crisis

During the European migrant crisis, migrant mothers and families had little understanding of the benefits of exclusive breastfeeding for the first six months of life. They perceived infant formula as the necessary and preferred sustenance for infant health [32, 36]. In addition, language and cultural differences [26, 30, 33, 34, 38], transit-linked issues such as minimal contact time with mothers and infants for assessment, care and counseling [26, 30, 33] and space constraints [30, 33, 38] made it even more difficult to provide adequate care for the refugees.

In Ukraine, responders experienced many of the same challenges identified above. The Code was violated when infant formula was distributed in the baby food assistance packages. In a population with a high pre-crisis mixed-feeding rate [25, 35, 47], the availability of infant formula further discouraged mothers to breastfeed. Misconceptions among Ukrainian mothers and families included a lack of understanding of the importance of continuing breastfeeding past 12 months of life [25, 35, 47]. In contrast with the European migrant crisis, the conflict in Ukraine resulted in a high number of Internally Displaced Persons who were not in constant movement, but relocated into camps [25]. IFE responses in Ukraine were designed for implementation in the camps where language and cultural differences were not barriers to the provision of infant feeding services to families [25, 35].

#### Context of natural disasters

Table 5 shows the eleven challenges that hindered optimal implementation of IFE programming. Many of these challenges were also present in the conflict settings and described above. The challenges unique to natural disasters were fewer (Table 3) and included damaged health infrastructure that limited access to healthcare or infant feeding support [44] and cases of infants requiring formula due to the deteriorating health or death of their mothers [41].

**Table 5:**
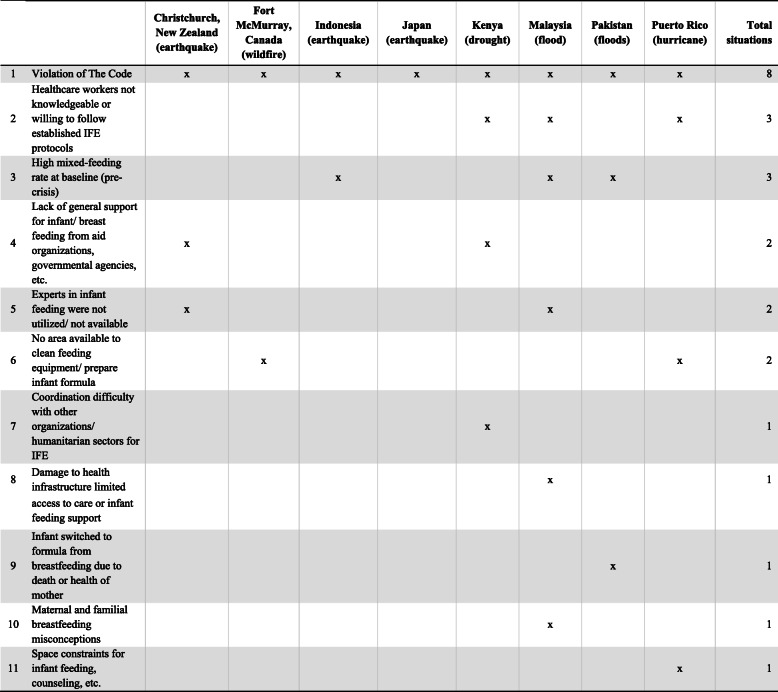
Challenges of IFE program implementation for responders due to natural disasters, by geographical area (*n* = 15)

The Code, The International Code of Marketing of Breastmilk Substitutes and subsequent WHA resolutions

IFE, infant feeding in emergencies

During the aftermath of the 2014 flood in Malaysia and the Christchurch earthquake in New Zealand, experts in infant feeding (e.g. breastfeeding counselors, midwives) were affected during and after the disaster themselves and unable to provide support [28, 44]. In some situations, families evacuating from natural disasters found that temporary shelters lacked areas to clean feeding equipment, prepare infant formula [20, 42, 43] or for mothers to feed their infants in privacy [42]. In Malaysia, damage to infrastructure such as health facilities and roads, limited families’ access to much needed infant feeding support [44].

### Barriers for mothers to breastfeed and/or provide breastmilk

#### Context of both conflicts and natural disasters

In addition to IFE program shortfalls, mothers faced many barriers to optimally breastfeed during conflict situations and natural disasters (Table 6). A major barrier was the lack of privacy or an environment unconducive to breastfeeding [19, 22, 26, 30, 32–34, 38, 42–44]. Other factors such as a mother’s limited fluid and nutrition intake [25, 26, 30, 33–35, 39, 44], stress and exhaustion [20, 25, 26, 28, 30, 32–35, 39, 43, 47] and a lack of time often due to constant movement [20, 26, 30, 33] also limited their ability to breastfeed. Additionally, many perceived that their milk supply was inadequate to satisfy infants [18, 23, 28, 30, 34, 39, 43]. In conflict settings, mothers were incorrectly advised by healthcare workers to stop breastfeeding if they had symptoms of a cold or increase in crying of their infant after feeding [25, 32, 35, 47]; similarly, after Hurricane Maria struck Puerto Rico, healthcare workers at public emergency shelters encouraged mothers to formula feed [43]. Furthermore, untargeted distribution of powdered infant formula in areas affected by the European migrant crisis [18, 26, 31] and during disasters in Japan and Puerto Rico provided mothers an alternative to breastfeeding [29, 43].

**Table 6:**
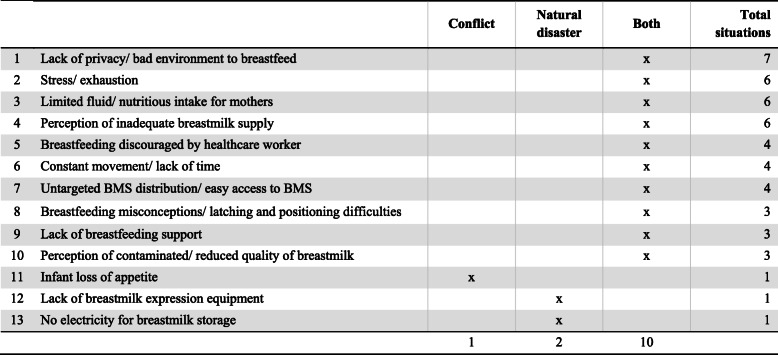
Summary: Barriers for mothers to breastfeed and/or provide breastmilk, by type of emergency (*n* = 25)

BMS, breastmilk substitute

#### Context of conflicts

Table 7 lists the barriers to breastfeeding caused by conflict situations. Only one barrier was unique to conflicts: mothers reported that their infants lost their appetite [34]. The eleven barriers identified were not specific to one geographical area and spanned across regions. Though the situation in Ukraine was distinct from the European migrant crisis, Ukrainian mothers faced many of the same barriers as refugee mothers in Jordan, Greece, the Balkans, and France.

**Table 7:**
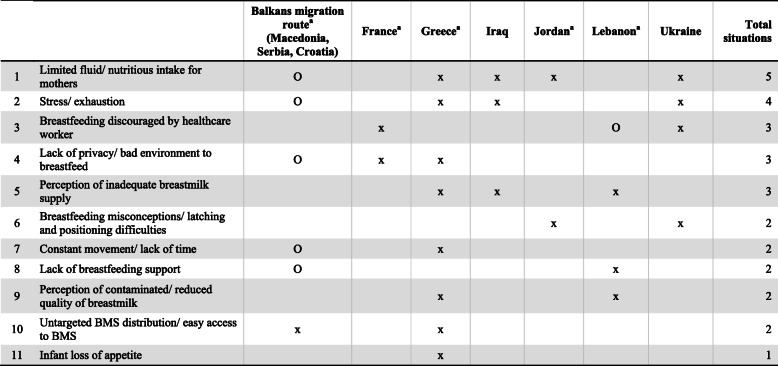
Barriers for mothers to breastfeed and/or provide breastmilk due to conflicts, by geographical area (*n* = 15)

KEY

x = Reported by mothers

O = Observed by healthcare workers or found through research

BMS, breastmilk substitute

^a^These countries were affected by the European migrant crisis

Overall, support for breastfeeding was limited, and mothers experiencing difficulties did not receive the help they needed [24, 33, 38]. Syrian refugee mothers in Jordan struggled with latching to the breast after healthcare workers introduced their infants to formula [31]. Mothers perceived their breastmilk to be ‘contaminated’ or of ‘reduced quality’ due to their own illness and medications, or bleeding nipples [18, 34].

#### Context of natural disasters

Breastfeeding barriers related to natural disasters are identified in Table 8. Two barriers were present after natural disasters that were not present in conflict contexts. Canadian mothers who experienced the Fort McMurray wildfire reported that the lack of breast pumps disrupted their typical feeding practices while they were displaced or on the move [20]. In Puerto Rico, the loss of electricity disrupted families’ usual practice of refrigerating expressed breastmilk [42].

**Table 8:**
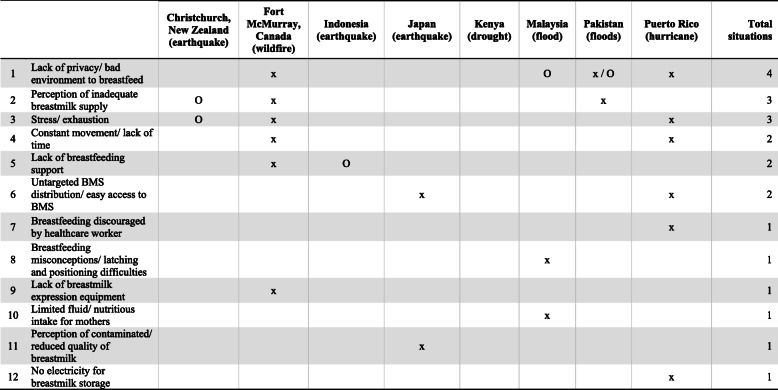
Barriers for mothers to breastfeed and/or provide breastmilk due to natural disasters, by geographical area (*n* = 15)

KEY

x = Reported by mothers

O = Observed by healthcare workers or found through research

BMS, breastmilk substitute

In several situations, breastfeeding support was lacking after disasters [20, 46]. Malaysian mothers believed they needed to discontinue breastfeeding if they were malnourished or their infant had diarrhea [44]. Furthermore, Japanese mothers worried that their breastmilk was contaminated from radiation exposure from the nuclear explosion; their concern led to breastfeeding interruptions and some mothers switched to formula [21, 37].

## Discussion

This review highlights the challenges responders faced when implementing IFE programs and identifies the barriers mothers faced breastfeeding or providing breastmilk during disasters in MICs and HICs. During the emergencies reviewed in this study, infant feeding support services, when available, did not meet the global standards and recommendations (see Table 1). The findings show that responders faced programmatic, systemic, resource and capacity constraints when implementing appropriate infant feeding interventions during disasters. These factors were also reported in a recently published article which audited the IFE preparedness in Australia [49].

In all the emergencies whether conflict or natural disaster-related, The Code was violated. This feature is particularly striking, as all affected countries (except Puerto Rico as a US territory) are Member States of the WHO and have endorsed The Code and agreed to adhere to its principles and standards. Donated and distributed infant formula was often labeled in languages foreign to the receiving population and without adequate preparation guidance. Infant formula was repeatedly distributed as part of the standardized assistance package regardless of mothers’ infant feeding practices. The uncontrolled distribution of donated infant formula not only undermined good breastfeeding practices and efforts of mothers, but also increased the health risk to infants. Mothers often had no access to electricity, gas, safe water, and necessary infant feeding supplies to hygienically prepare infant formula.

Implementation of IFE programs can be logistically complex. In instances where a mother was not exclusively breastfeeding and there was a lack of resources and space to hygienically prepare bottles with powdered infant formula, the appropriate BMS is Ready-to-Use Infant Formula. However, management of artificial feeding continued to be a challenge due to funding, capacity, securing supply and estimating demand, and ensuring labels were language and culturally appropriate.

During disasters, mothers who chose to breastfeed found displacement created additional challenges. It was difficult to find comfortable, private places and the time to breastfeed. Stress and disruption of daily routine posed new challenges for breastfeeding women. Displacement, together with the loss of health and social infrastructure, often led to a breakdown of networks of family and friends that normally supported mothers with breastfeeding. It should be noted that while the disasters created new challenges for mothers, some pre-existing infant feeding practices and misconceptions also deterred mothers from pursuing the recommended infant feeding practices during disasters.

Few healthcare workers or responders were trained to offer breastfeeding counseling and infant feeding management. In some situations, healthcare workers inadvertently undermined mothers’ efforts to breastfeed by providing incorrect recommendations to switch to formula feeding under conditions where the infrastructure to safely prepare the infant formula was inadequate. This practice was compounded by existing misconceptions among mothers and their families which also discouraged them to breastfeed during emergencies. This situation was particularly prevalent among the Syrian refugee population where the mixed-feeding rate was already high before the crisis resulting in a further decrease of breastfeeding [26, 30, 32].

Outside of the emergency context, the World Breastfeeding Trends Initiative (WBTi), led by the Breastfeeding Promotion Network of India, assists countries in assessing and monitoring their implementation of the Global Strategy of Infant and Young Child Feeding using 15 indicators [50]. A study that analyzed WBTi reports from 18 European countries concluded that the key problem is the lack of proper national policies, programs and coordination to protect children’s right to optimal nutrition, even in everyday settings [51]. Despite MICs and HICs having more resources available during disasters, adequate IFE programming is still not guaranteed. Of the 18 countries in Europe that participated in the WBTi assessments, North Macedonia is the only country that has a national policy that includes all the basic components of the IFE Operational Guidance [51]. Advocacy on the lifesaving impact of appropriate infant feeding practices is urgently needed, especially in the emergency context. The problem is not the lack of a comprehensive guide on IFE programming, but a lack of national IFE preparedness and response plans and capacity when emergencies arise. Sensitization and adherance to key documents (listed in Table 1), such as The Code and the IFE Operational Guidance are critical for successful IFE response in disasters.

A study in the 2016 Lancet Breastfeeding series proposed six actions points to support women and their families to breastfeed optimally [52]. The findings of this study confirm the urgent need for action and investment by governments from MICs and HICs, UN agencies, and international and national aid organizations to ensure full compliance with The Code, that emergency responders are trained and equipped with relevant skills and resources to provide infant feeding support, and that safe spaces for women and infants are created and equipped to ensure that women and their infants are able to practice appropriate infant feeding during emergencies.

IFE programming should be multi-sectoral with interventions and strategies mainstreamed among sectors. Interventions should include breastfeeding counseling as part of the minimum package of health services for women with infants. Processes to prioritize pregnant women and women with young children during food and non-food distributions, and to provide women with infant’s priority access to water and sanitation facilities are also needed.

The internationally recognized Sphere Handbook of humanitarian standards and principles calls on all humanitarian agencies to ensure that actions and interventions follow the “do no harm” principle which is imperative in times of crises [53]. These standards and principles are based on the recognition that the aid provided may lead to unintended outcomes that aggravate the conditions of the affected populations. In the context of infant feeding, large donations of infant formula are often received as part of the emergency response. While the intentions are generally good, there is a lack of awareness that such donations can do more harm than good as there are neither basic infrastructure nor adequate conditions to reduce the risks linked to the preparation of infant formula. In these emergencies, such donations should always be targeted based on individual assessments and accompanied with substantial input regarding infrastructure and counseling [54].

The authors recommend that governments, agencies and organizations involved in disaster preparedness and response commit to endorsing and funding policies and programs that uphold the recommendations of The Code [7], the WHO/UNICEF Global Strategy for Infant and Young Child Feeding [9] and the IFE Operational Guidance [11], specifically to

1. Include IFE as a key component of health and nutrition emergency preparedness and response plans (at national and sub-national levels) to ensure protection, promotion and support for breastfeeding and management and support for the non-breastfed infants (i.e. infant formula dependent infants).
2. Integrate relevant IFE elements across other sectors (i.e. Child Protection, Water, Sanitation, and Hygiene [WASH], Food Security and Livelihood, Health, Shelter) by creating more contact points with women and infants to address misconceptions around infant feeding, and to identify women with breastfeeding difficulties and concerns that may need infant feeding support.
3. Invest in building IFE capacity of responders and health and nutrition agencies to provide timely, appropriate, resourced and skilled IFE responses.
4. Ensure a minimum set of IFE indicators are integrated in the emergency information system and included in the decision making process (i.e. WBTi indicators) [50].
5. Allocate resources for IFE in emergency response to support and meet the specific needs of mothers and infants.

### Limitations

This review was not a systematic review of the topic but a scoping review that focused on a wide variety of literature. However, the documented experiences and lessons learned in the grey literature provided a wealth of information otherwise not mentioned in peer-reviewed research.

## Conclusions

The protection, promotion, and support of breastfeeding during disasters has been a major global effort since 1981. There are sufficient guidelines that provide evidence-based recommendations for optimal infant feeding practices, even in disaster settings. The review provides valuable insight to which IFE challenges persist despite existing guidelines, and uncovers a lack of IFE preparedness and response capacity at the national level of various MICs and HICs leaving infants at risk for serious health and developmental consequences. It is critical for national governments, UN agencies and international and national aid organizations to endorse and adapt global recommendations to develop national IFE plans and programs with emphasis on capacity building for disaster responders.

## Data Availability

Not applicable.

## Abbreviations

BMS: Breastmilk Substitute
IFE: Infant Feeding in Emergencies
HIC: High-income countries
MIC: Middle-income countries
The Code: The International Code of Marketing of Breastmilk Substitutes and subsequent WHA resolutions
UN: United Nations
WASH: Water, Sanitation, and Hygiene
WBTi: World Breastfeeding Trends Initiative
WHA: World Health Assembly
WHO: World Health Organization

## References

[1] Victora CG, Bahl R, Barros AJD, França GVA, Horton S, Krasevec J, Murch S, Sankar MJ, Walker N, Rollins NC (2016). Breastfeeding in the 21st century: epidemiology, mechanisms, and lifelong effect. Lancet..

[2] Horta BL, Bahl R, Martines JC, Victora CG. Evidence on the long-term effects of breastfeeding: systematic reviews and meta-analyses. WHO. 2007:1–52. https://apps.who.int/iris/bitstream/handle/10665/43623/9789241595230_eng.pdf?sequence=1&isAllowed=y.

[3] Andersson N, Paredes-Sols S, Legorreta-Soberanis J, Cockcroft A, Sherr L (2010). Breast-feeding in a complex emergency: four linked cross-sectional studies during the Bosnian conflict. Public Health Nutr.

[4] Creek T, Kim A, Lu L, Bowen A, Masunge J, Arvelo W (2010). Hospitalization and mortality among primarily nonbreastfed children during a large outbreak of diarrhea and malnutrition in Botswana, 2006. J Acquir Immune Defic Syndr.

[5] Hipgrave DB, Assefa F, Winoto A, Sukotjo S (2012). Donated breast milk substitutes and incidence of diarrhoea among infants and young children after the may 2006 earthquake in Yogyakarta and Central Java. Public Health Nutr.

[6] Lipton E, Drew C, Shane S. Breakdowns Marked Path From Hurricane to Anarchy [Internet]. New York Times. New York; 2005 [cited 2020 Dec 28]. Available from: https://www.nytimes.com/2005/09/11/us/nationalspecial/breakdowns-marked-path-from-hurricane-to-anarchy.html

[7] World Health Organization. International Code of Marketing of World Health Organization. Geneva; 1981.

[8] World Health Organization. Nutrition: code and subsequent resolutions [Internet]. [cited 2019 Jun 12]. Available from: https://www.who.int/nutrition/netcode/resolutions/en/

[9] World Health Organization, UNICEF. Global strategy for infant and young child feeding. Geneva; 2003.

[10] IFE Core Group. Infant and Young Child Feeding in Emergencies: Operational Guidance for Emergency Relief Staff and Programme Managers- Version 2.1. Oxford; 2007.

[11] IFE Core Group. Infant and Young Child Feeding in Emergencies: Operational Guidance for Emergency Relief Staff and Programme Managers- Version 3.0. Oxford; 2017.

[12] World Health Assembly. Seventy-First World Health Assembly: Infant and Young Child Feeding. Geneva: World Health Organization; 2018. p. 3.

[13] World Health Organization. Guiding principles for feeding infants and young children. Geneva; 2004.

[14] World Health Organization. Global Nutrition Policy Review 2016-2017. Geneva; 2018.

[15] The World Bank. World Bank Country and Lending Groups [Internet]. 2019 [cited 2019 Mar 1]. Available from: https://datahelpdesk.worldbank.org/knowledgebase/articles/906519-world-bank-country-and-lending-groups

[16] World Vision International. What is a humanitarian disaster? [Internet]. [cited 2019 Apr 12]. Available from: https://www.wvi.org/disaster-management/what-humanitarian-disaster

[17] Humanitarian Coalition. What is a Humanitarian Emergency? [Internet]. [cited 2019 Apr 12]. Available from: https://www.humanitariancoalition.ca/what-is-a-humanitarian-emergency

[18] Akik C, Ghattas H, Filteau S, Knai C (2017). Barriers to breastfeeding in Lebanon: a policy analysis. J Public Health Policy.

[19] Bukhari SIA, Rizvi SH. Impact of floods on women: with special reference to flooding experience of 2010 flood in Pakistan. Journal of Geography & Natural Disasters. 2015;5(2):1–5.

[20] DeYoung SE, Chase J, Branco MP, Park B (2018). The effect of mass evacuation on infant feeding: the case of the 2016 Fort McMurray wildfire. Matern Child Health J.

[21] Kyozuka H, Yasuda S, Kawamura M, Nomura Y, Fujimori K, Goto A, Yasumura S, Abe M (2016). Impact of the great East Japan earthquake on feeding methods and newborn growth at 1 month postpartum: results from the Fukushima health management survey. Radiat Environ Biophys.

[22] Maheen H, Hoban E (2017). Rural women’s experience of living and giving birth in relief camps in Pakistan. PLOS Curr Disasters.

[23] Sadia H, Iqbal MJ, Ahmad J, Ali A, Ahmad A (2016). Gender-sensitive public health risks and vulnerabilities’ assessment with reference to floods in Pakistan. Int J Disaster Risk Reduct.

[24] Shaker-Berbari L, Ghattas H, Symon AG, Anderson AS (2018). Infant and young child feeding in emergencies: organisational policies and activities during the refugee crisis in Lebanon. Matern Child Nutr.

[25] Summers A, Bilukha OO (2018). Suboptimal infant and young child feeding practices among internally displaced persons during conflict in eastern Ukraine. Public Health Nutr.

[26] Svoboda A. Retrospective qualitative analysis of an infant and young child feeding intervention among refugees in Europe. Field Exchange. 2017;(55):85.

[27] Codjia P, Volege M, Le MT, Donnelly A, Sesay FF, Senesie JV, et al. Enhancing infant and young child feeding in emergency preparedness and response in East Africa: capacity mapping in Kenya, Somalia and South Sudan. Field Exchange. 2018;(57):35.

[28] Hargest-Slade AC, Gribble KD (2015). Shaken but not broken: supporting breastfeeding women after the 2011 Christchurch New Zealand earthquake. Breastfeed Rev.

[29] Hongo H. Breastfeeding support after the Great East Japan earthquake [Internet]. WABA MSTF E-newsletter. 2012 [cited 2019 Jan 26]. p. 5–6. Available from: http://www.waba.org.my/pdf/mstfnl_v10n1_eng.pdf

[30] Le MT, Prudhon C, Mayer A-M, Gayford M. Infant and young child feeding in Greece: Save the Children’s experience. Humanitarian Exchange. 2016:41–3.

[31] Mboya S. Artificial feeding in emergencies: experiences from the ongoing Syrian crisis. Field Exchange. 2014;(48):164.

[32] Middlemiss L. Infant feeding in the refugee crisis [internet]. Association of Breastfeeding Mothers. 2018;(48):164. Available from: https://abm.me.uk/wp-content/uploads/2018/03/mag4-feature.pdf.

[33] Modigell I, Fernandes C, Gayford M. Save the Children’s IYCF-E rapid response in Croatia. Field Exchange. 2016;102(52):106.

[34] Prudhon C. Assessment of infant and young child feeding practices among refugees on Lesvos Island, Greece. London: Save the Children; 2016.

[35] Summers A, Bilukha O (2015). Emergency infant and young child feeding assessment among internally displaced persons-Kharkiv, Dnipropetrovsk, and Zaporizhia.

[36] Fänder G, Frega M. Responding to nutrition gaps in Jordan in the Syrian refugee crisis: infant and young child feeding education and malnutrition treatment. Field Exchange. 2014;(48):82.

[37] Binns CW, Lee MK, Tang L, Yu C, Hokama T, Lee A (2012). Ethical issues in infant feeding after disasters. Asia Pacific J Public Health.

[38] Dimitrievska V. Breastfeeding among Refugee Mothers on the Balkan Route [Internet]. 2016 [cited 2019 Jan 25]. Available from: https://anthrolactology.com/2016/02/25/breastfeeding-among-refugee-mothers-on-the-balkan-route/

[39] Haidar MK, Ben FJ, Saim M, Morton N, Defourny I (2017). Severe malnutrition in infants displaced from Mosul, Iraq. Lancet Glob Heal.

[40] Hirani SAA (2014). Vulnerability of internally displaced children in disaster relief camps of Pakistan: issues, challenges, and way forward. Early Child Dev Care.

[41] Hirani SAA, Kenner C (2011). International column: effects of humanitarian emergencies on newborn and infants’ health in Pakistan. Newborn Infant Nurs Rev.

[42] Santaballa Mora LM (2018). Challenges of infant and child feeding in emergencies: the Puerto Rico experience. Breastfeed Med.

[43] Staley R. Breastfeeding for the apocalypse [Internet]. Corporate Knights. 2018 [cited 2019 Jan 25]. Available from: https://www.corporateknights.com/voices/roberta-staley/breastfeeding-for-the-apocalypse-15161652/

[44] Sulaiman Z, Mmed NM, Med F, Alina T, Ismail T, Johari Bsc N (2016). Infant feeding concerns in times of natural disaster: lessons learned from the 2014 flood in Kelantan, Malaysia. Asia Pac J Clin Nutr.

[45] Ververs M, McGrath M, Gribble K, Fernandes C, Kerac M, Stewart RC (2018). Infant formula in Iraq: part of the problem and not a simple solution. Lancet Glob Heal.

[46] IBU Foundation. Influx of Baby Food Supplies Swamped Central Sulawesi Emergency Camps in Indonesia, Undermining Breastfeeding and Optimal Infant and Young Child Feeding [Internet]. 2018 [cited 2019 Jan 25]. Available from: https://www.ibfan-icdc.org/influx-of-baby-food-supplies-swamped-central-sulawesi-emergency-camps-in-indonesia/

[47] World Food Program. Only 1 in 4 internally displaced infants is exclusively breastfed in Ukraine. UNICEF and WFP call to protect and promote breastfeeding among conflict-affected mothers in eastern Ukraine [Internet]. 2015 [cited 2019 Jan 25]. Available from: https://www.wfp.org/news/only-1-4-internally-displaced-infants-exclusively-breastfed-ukraine-unicef-and-wfp

[48] World Health Organzation. Definition of key terms [Internet]. 2013 [cited 2020 Aug 28]. Available from: https://www.who.int/hiv/pub/guidelines/arv2013/intro/keyterms/en/

[49] Gribble K, Peterson M, Brown D (2019). Emergency preparedness for infant and young child feeding in emergencies (IYCF-E): an Australian audit of emergency plans and guidance. BMC Public Health.

[50] World Breastfeeding Trends Initiative (WBTi). About WBTi [Internet]. [cited 2020 Aug 28]. Available from: https://www.worldbreastfeedingtrends.org/p/what-is-wbti

[51] Zakarija-Grković I, Cattaneo A, Bettinelli ME, Pilato C, Vassallo C, Borg Buontempo M (2020). Are our babies off to a healthy start? The state of implementation of the global strategy for infant and young child feeding in Europe. Int Breastfeed J.

[52] Rollins NC, Bhandari N, Hajeebhoy N, Horton S, Lutter CK, Martines JC, Piwoz EG, Richter LM, Victora CG (2016). Why invest, and what it will take to improve breastfeeding practices?. Lancet..

[53] Sphere. The Sphere Handbook: Humanitarian Charter and Minimum Standards in Humanitarian Response. Geneva; 2018.

[54] IBFAN-ICDC. The Code and infant feeding in emergencies [Internet]. Penang, Malaysia; 2009 [cited 2020 Aug 20]. Available from: https://www.unhcr.org/uk/protection/health/4b751edf9/icdc-focus-code-infant-feeding-emergencies.html

